# Supplementary material for: The interactome of intact mitochondria by cross-linking mass spectrometry provides evidence for coexisting respiratory supercomplexes
Source: Mol Cell Proteomics. 2017 Dec 8;17(2):216–32. doi: 10.1074/mcp.RA117.000470 (PMC5795388; doi:10.1074/mcp.RA117.000470)
Supplement: Supplemental Data [file supp_RA117.000470_133922_0_supp_23978_fzffwf.zip › spectra_annotation/mito_spectra_annotation/19-1-4-1-3-1.pdf]

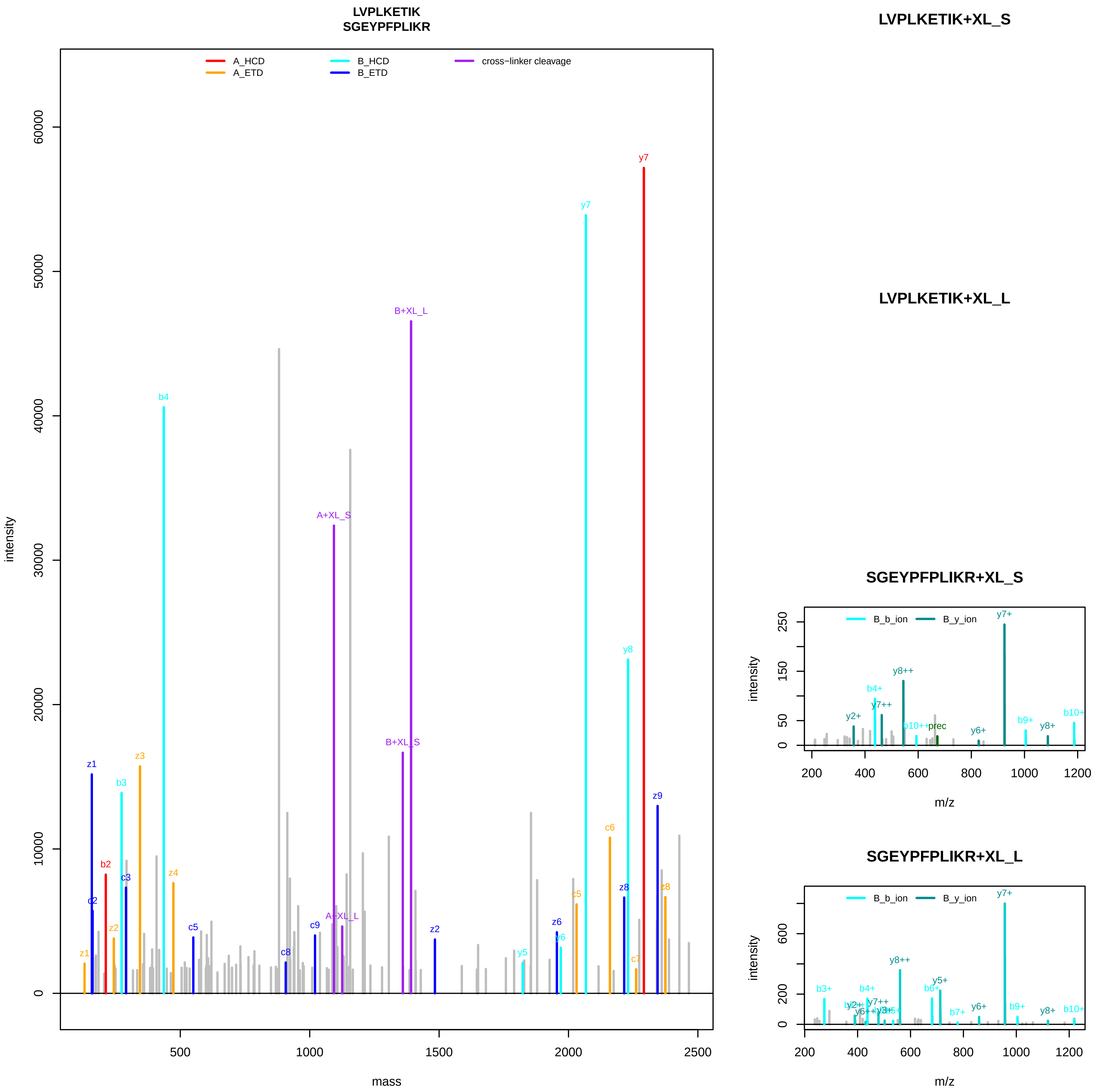

LVPLKETIK
SGEYPFPLIKR
A_HCD
A_ETD
B_HCD
B_ETD
cross−linker cleavage
intensity
mass
0
10000
20000
30000
40000
50000
60000
500
1000
1500
2000
2500
y7
B+XL_L
A+XL_S
B+XL_S
A+XL_L
b4
b3
b2
z1
z2
z3
z4
z6
z8
z9
c2
c3
c5
c6
c7
c8
c9
y5
y6
y8
LVPLKETIK+XL_S
LVPLKETIK+XL_L
SGEYPFPLIKR+XL_S
SGEYPFPLIKR+XL_L
B_b_ion
B_y_ion
m/z
0
50
150
250
200
400
600
800
1000
1200
y7+
y8++
b4+
y7++
y2+
b10++
prec
y6+
b9+
y8+
b10+
b3+
y5+
b6+
b7+
